# Supplementary material for: Pre-Administration of PLX-R18 Cells Protects Mice from Radiation-Induced Hematopoietic Failure and Lethality
Source: Genes (Basel). 2022 Sep 28;13(10):1756. doi: 10.3390/genes13101756 (PMC9601513; doi:10.3390/genes13101756)
Supplement: Supplementary file 1 [file genes-13-01756-s001.zip › genes-1924372-supplementary.pdf]

Supplementary figures

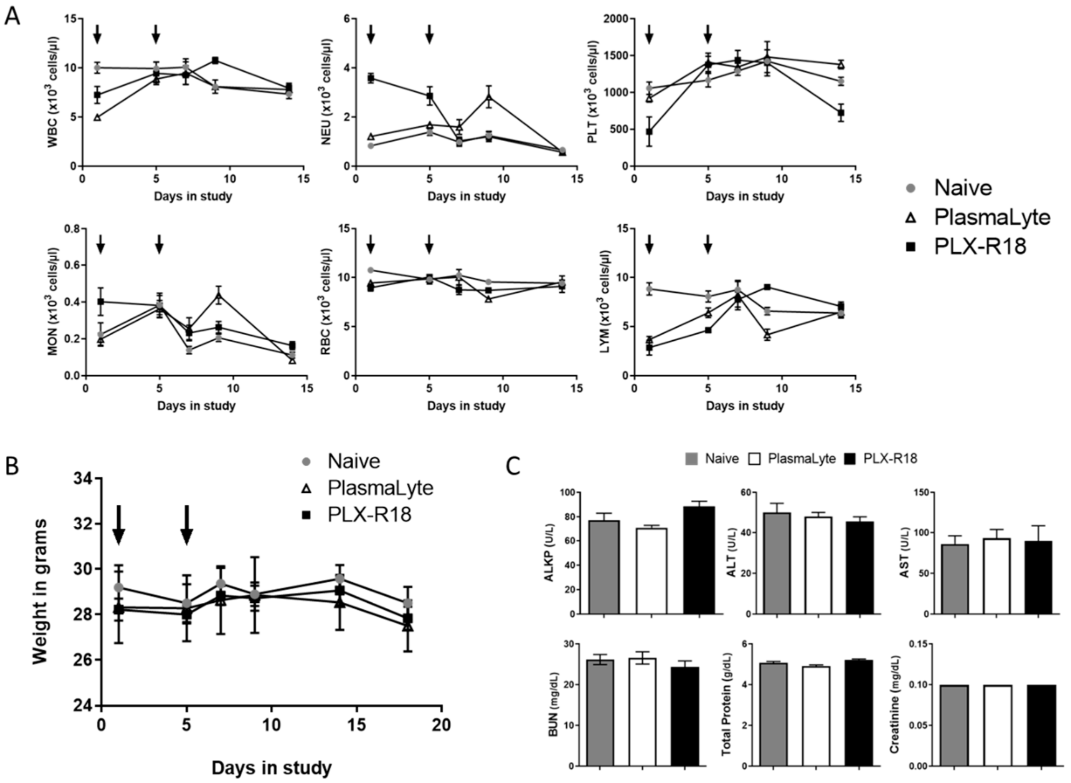

Figure S1. Basic safety study with PLX-R18. (A) Peripheral blood cell counts compared between naïve (●), PlasmaLyte (Δ) and PLX-R18 (■) groups. (B) Body weights of the animals in naïve (●), PlasmaLyte (Δ) and PLX-R18 (■) groups for the duration of the study. (C) Results of the serum levels of the renal and hepatic panel. The weights and CBC were done on the same days. Data represented are mean ± standard error of the mean (SEM) for n = 5 mice.

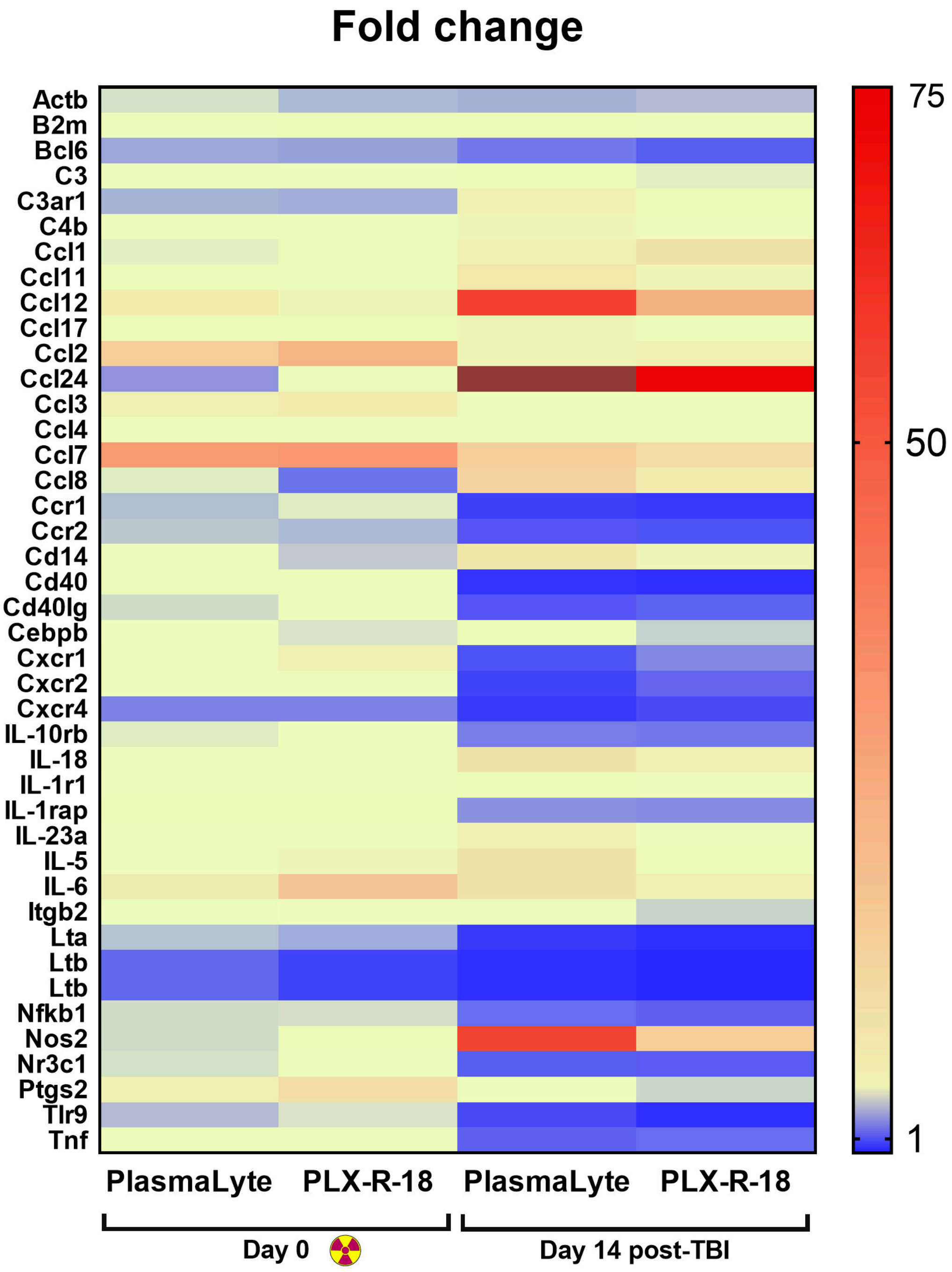

Figure S2. PLX-R18 modulates various genes from the autoimmune pathway. Spleen RNA was subjected to qRT-PCR to measure levels of expression of several genes of the auto immune pathway. The heat-map represents the data for 42 genes expressed in four irradiated groups of animals on day 0 treated with either PlasmaLyte (Rd0V) or PLX-R18 (Rd0D) and day 14 treated with either PlasmaLyte (Rd14V) or PLX-R18 (Rd14D).
